# Comparative Analysis of Early Versus Traditional Feeding Protocols in Elective Lower Segment Cesarean Sections

**DOI:** 10.7759/cureus.68367

**Published:** 2024-09-01

**Authors:** Nikita, Seema Saran, Meenakshi Singh

**Affiliations:** 1 Obstetrics and Gynaecology, Government Medical College, Budaun, Budaun, IND; 2 Obstetrics and Gynaecology, Lady Hardinge Medical College, Delhi, IND

**Keywords:** comparative study, patient’s satisfaction, lower segment caesarean section (lscs), traditional postoperative oral feeding (tof), early feeding

## Abstract

Background

Postoperative care following elective lower segment cesarean section (LSCS) traditionally involves delayed oral feeding. However, recent evidence suggests that early feeding may enhance recovery and improve patient outcomes. This study aimed to compare the recovery outcomes of elective LSCS patients between early feeding and traditional feeding protocols.

Methods

This prospective, comparative study was conducted at the Department of Obstetrics and Gynecology, Government Medical College (GMC) Budaun, over nine months. Women aged 18 to 40 years undergoing elective LSCS with singleton pregnancies and gestational ages between 37 and 42 weeks were included. Participants were randomized into two groups: the early feeding group (EFG) and the traditional feeding group (TFG). The EFG received oral intake as early as two hours post-surgery, progressing to a regular diet within six to eight hours. The TFG followed standard postoperative protocols, beginning oral intake after 12-24 hours. Primary outcomes included time to return of bowel function and length of hospital stay. Secondary outcomes were patient satisfaction and complication rates. Data were analyzed using SPSS version 22.0 (IBM Corp., Armonk, NY), with p-values < 0.05 considered statistically significant.

Results

The study included 94 participants (EFG: n = 44, TFG: n = 50). The EFG showed significantly faster return of bowel function, with time to first flatus (23.9 ± 6.2 vs. 34.1 ± 8.8 hours, p < 0.0001) and first stool (54.6 ± 8.5 vs. 91.3 ± 12.3 hours, p < 0.0001). Length of hospital stay was shorter in the EFG (4.3 ± 1.1 vs. 6.7 ± 1.4 days, p < 0.0001). Visual analog scale (VAS) scores before discharge were higher in the EFG (94.4 ± 8.7 vs. 81.4 ± 9.5, p < 0.0001), indicating greater patient satisfaction. Complication rates, including nausea, vomiting, abdominal distension, and wound infections, did not differ significantly between groups.

Conclusion

Early feeding post-elective LSCS significantly enhances recovery, as evidenced by quicker return of bowel function, reduced hospital stay, and higher patient satisfaction without increasing complication rates. These findings support revising postoperative care protocols to incorporate early feeding strategies.

## Introduction

Lower segment cesarean section (LSCS) is a common surgical procedure performed to deliver a baby when vaginal delivery poses risks to the mother or child [1]. Despite being a routine operation, postoperative care and recovery strategies for LSCS patients remain a critical area of interest [2]. The timing of postoperative feeding is a significant component of post-surgical care, influencing recovery outcomes such as gastrointestinal function, pain management, and overall patient well-being [3].

Traditionally, postoperative care for LSCS patients has followed a conservative approach, delaying oral intake until bowel function returns, usually marked by the passage of flatus. This method aims to reduce the risk of postoperative ileus, nausea, and vomiting [4]. However, this delay in feeding can lead to patient discomfort, delayed mobilization, and extended hospital stays [5].

In contrast, recent studies suggest that early oral feeding, initiated within hours after surgery, may expedite recovery without increasing complications [6]. Enhanced recovery after cesarean (ERAC) provides an evidence-based system to improve maternal outcomes, functional recovery, maternal-infant bonding, and patient experience. ERAC involves the multidisciplinary efforts of the anaesthesiologist, obstetrician, nurses, hospital, and patient [7].

Despite the emerging evidence, there remains a lack of consensus on the optimal timing of postoperative feeding for LSCS patients. Previous research has produced mixed results, and variations in study design, patient populations, and definitions of "early" versus "traditional" feeding complicate the generalization of findings [8-10]. Therefore, a comprehensive comparison of recovery outcomes between early and traditional feeding groups in elective LSCS patients is necessary to inform clinical practice and improve patient care.

This study aimed to assess the impact of early oral intake on the post-cesarean patient recovery experience using visual analog scale (VAS). Also, by evaluating parameters such as time to passage of flatus, feces, first urination after catheter removal, and discharge from the hospital, this research seeks to provide evidence-based recommendations for optimizing postoperative feeding strategies in this population.

## Materials and methods

Study design

This study was a prospective, comparative study, which was designed to compare the recovery outcomes of patients in the early feeding group (EFG) with those in the traditional feeding group (TFG) following elective LSCS (under spinal anesthesia). The study was conducted at the Department of Obstetrics and Gynecology, Government Medical College (GMC) Budaun, for nine months between September 2023 and May 2024. The study protocol was approved by the GMC Institutional Review Board (IRB/GMCB/01/Aug/2023). Written informed consent was obtained from all participants before enrollment in the study.

Study population

The inclusion criteria for the study were as follows: women aged 18 to 40 years, undergoing elective LSCS, with a singleton pregnancy and gestational age between 37 and 42 weeks, no significant comorbidities such as diabetes mellitus or hypertension, and the ability to provide informed consent. The exclusion criteria included emergency LSCS, pre-existing gastrointestinal disorders (Crohn's disease and irritable bowel syndrome), severe maternal or fetal complications (pre-eclampsia and fetal distress), and participation in another clinical trial within the last 30 days. Additionally, participants who were on any medication that aids in bowel movement were excluded to avoid confounding effects on the primary outcomes related to gastrointestinal function and recovery.

Randomization and group allocation

Randomization and group allocation were performed using a computer-generated randomization schedule to ensure allocation concealment. Sequentially numbered, sealed, opaque envelopes were used to assign participants to one of the two study groups immediately after surgery. Patients in the EFG received a diet of their choice (mostly tea and biscuits) served during postoperative hour 2 or whenever bowel sounds appeared. If tolerated, these patients progressed to a regular diet starting six to eight hours post-surgery [9,10]. In contrast, patients in the TFG followed the standard postoperative protocol of nil per os (NPO) for the first 12-24 hours, followed by a gradual reintroduction of liquids and solids as tolerated.

Study interventions

Early Feeding Group (EFG)

In the EFG, feeding was initiated orally, depending on the patient's condition and the surgeon’s discretion. The initial diet primarily comprised tea and biscuits, and patients progressed to a regular diet starting six to eight hours post-surgery if tolerated. Monitoring of patients in this group included assessing gastrointestinal tolerance, characterized by the absence of significant nausea, vomiting, and abdominal distension, along with clinical signs of bowel function such as bowel sounds and passage of flatus.

Traditional Feeding Group (TFG)

In the TFG, patients remained NPO for the first 12-24 hours post-surgery. Clear liquids were introduced thereafter, with a gradual transition to a soft diet and eventually a regular diet over the next 48-72 hours, which is determined by individual tolerance and clinical assessment. Monitoring in this group included daily evaluations of gastrointestinal function and overall recovery to ensure safe and effective postoperative management.

Data collection

Data were systematically collected from patient's medical records and through direct patient interviews. Demographic information, such as age, sex, residence (rural and urban), body mass index (BMI), and comorbidities (e.g., diabetes and hypertension), was extracted from the patient's medical records. Details regarding the type and duration of surgery, perioperative medication use, and gastrointestinal complications (including symptoms and signs) were also obtained from the medical records. Additional information, such as patient satisfaction and recovery milestones, was collected through structured questionnaires administered during the postoperative period and at follow-up visits.

Outcome measures

Visual Analogue Scale (VAS) was used to assess satisfaction on the 10th day of surgery. The VAS is a 100-mm long scale with descriptions at both ends, where 0= lack of satisfaction about the timing of the meal after surgery and 100 = full satisfaction about the timing of the meal after surgery [11].

Recovery, in the context of this study, refers to the evaluation of postoperative progress and well-being following elective LSCS, using a comprehensive set of parameters. These parameters include the time to pass flatus and feces, which represent the duration elapsed from the completion of surgery until the patient first passes gas and stool, indicative of the restoration of gastrointestinal function post-LSCS. Additionally, the time to first mobilization, reflecting the period from the end of the surgical procedure to the patient's first physical mobilization such as sitting up, standing, or walking, offers insights into early postoperative ambulation and recovery of mobility. The time to first void after catheter removal, indicating the time taken for the patient to urinate spontaneously after the removal of the urinary catheter, provides information on the restoration of normal bladder function and urinary continence post-surgery. Finally, the length of hospital stay, measuring the duration of the hospitalization period from the completion of surgery to the time of discharge, offers valuable insights into the overall recovery trajectory and readiness for discharge following elective LSCS. Monitoring for gastrointestinal complications included documenting symptoms and signs every four hours. Follow-up assessments were conducted via outpatient visits or telephone interviews at 30 days postoperatively to capture any late complications or readmissions.

Statistical analysis

Descriptive statistics were used to summarize the baseline characteristics and outcomes of the study population. Continuous variables were presented as mean ± standard deviation (SD), and categorical variables were presented as frequencies and percentages. The descriptive variables included demographic information such as age (years), residence (rural or urban), and BMI (kg/m²) as well as clinical and perioperative data such as gestational age (weeks), comorbidities (presence of diabetes, hypertension, or other significant medical conditions), duration of surgery (minutes), and perioperative medication use (use of medications during the perioperative period). Postoperative recovery outcomes included VAS (before discharge, satisfaction levels measured using the VAS before discharge), time to first feed (hours), time to second feed (hours), time to first flatus (hours), time to first stool (hours), time to first mobilization (hours), time to first void after catheter removal (hours), length of hospital stay (days), and patient satisfaction levels (classified as very satisfied, satisfied, neutral, dissatisfied, and very dissatisfied). Complications assessed included nausea, vomiting, abdominal distension, ileus, wound infection, urinary tract infection, deep vein thrombosis, and readmissions within 30 days postoperatively. Continuous variables were compared between the EFG and TFG using the student’s t-test for normally distributed data or the Mann-Whitney U test for non-normally distributed data, while categorical variables were analyzed using the chi-square test or Fisher’s exact test, as appropriate. A p-value of <0.05 was considered statistically significant. Statistical analysis was conducted using SPSS version 22.0 (IBM Corp., Armonk, NY).

Ethical considerations

This study adhered to the ethical principles outlined in the Declaration of Helsinki. Informed consent was obtained from all participants after providing them with detailed information about the study's purpose, procedures, potential risks, and benefits. Participants were assured of their right to withdraw from the study at any time without affecting their medical care.

## Results

A total of 94 participants were enrolled in the study (44 participants in EFG and 50 participants in TFG). While there were no significant differences in age (EFG: 26.9 ± 3.5 years vs. TFG: 27.9 ± 3.8 years, p = 0.189) or BMI (EFG: 26.2 ± 2.4 kg/m² vs. TFG: 26.0 ± 2.6 kg/m², p = 0.701) between the groups, a higher proportion of urban residents was observed in the EFG compared to the TFG (81.8% vs. 96.0%, p = 0.026). Gestational age at surgery, prevalence of comorbidities, duration of surgery, and perioperative medication use showed no statistically significant differences between the groups (p > 0.05 for all) (Table 1).

**Table 1:** Demographic and clinical characteristics of participants in the early feeding group (EFG) and traditional feeding group (TFG) BMI: Body mass index. Statistically significant for p < 0.05.

Characteristics	Early Feeding Group (EFG) (n = 44)	Traditional Feeding Group (TFG) (n = 50)	p-value
	Number (%)/mean ± SD		
Age (years)	26.9 ± 3.5	27.9 ± 3.8	0.189
Residence			
Rural	8 (18.2%)	2 (40.0%)	0.026
Urban	36 (81.8%)	48 (96.0%)	
BMI (kg/m²)	26.2 ± 2.4	26.0 ± 2.6	0.701
Gestational age (weeks)	37.4 ± 1.2	37.8 ± 1.3	0.126
Comorbidities	4 (9.1%)	5 (10.0%)	0.881
Duration of surgery (minutes)	45.8 ± 5.2	46.3 ± 5.5	0.653
Perioperative medication use	42 (95.5%)	47 (94.0%)	0.749

The outcomes for the EFG and TFG demonstrated significant benefits for the EFG. The VAS scores before discharge were higher in the EFG (94.4 ± 8.7) compared to the TFG (81.4 ± 9.5, p < 0.0001), indicating greater patient satisfaction. The EFG had a significantly shorter mean time to the first feed (2.2 ± 0.8 hours vs. 23.9 ± 2.1 hours, p < 0.0001) and the second feed (10.4 ± 1.2 hours vs. 34.2 ± 3.4 hours, p < 0.0001). The EFG also experienced a quicker return of bowel function, with a mean time to first flatus of 23.9 ± 6.2 hours compared to 34.1 ± 8.8 hours in the TFG (p < 0.0001) and first stool of 54.6 ± 8.5 hours versus 91.3 ± 12.3 hours in the TFG (p < 0.0001). Additionally, the EFG had a shorter time to first mobilization (23.6 ± 6.3 hours vs. 32.9 ± 8.6 hours, p < 0.0001) and a shorter hospital stay (4.3 ± 1.1 days vs. 6.7 ± 1.4 days, p < 0.0001). There was no significant difference in the time to first void after catheter removal between the groups (p = 0.519) (Table 2).

**Table 2:** Outcomes of interest for the early feeding group (EFG) and the traditional feeding group (TFG) VAS: Visual analog scale. Statistically significant for p < 0.05.

Outcome	Early Feeding Group (EFG) (n = 44)	Traditional Feeding Group (TFG) (n = 50)	p-value
	Mean ± SD		
VAS (Before discharge)	94.4 ± 8.7	81.4 ± 9.5	<0.0001
Time to first feed (hours)	2.2 ± 0.8	23.9 ± 2.1	<0.0001
Time to second feed (hours)	10.4 ± 1.2	34.2 ± 3.4	<0.0001
Time to first flatus (hours)	23.9 ± 6.2	34.1 ± 8.8	<0.0001
Time to first stool (hours)	54.6 ± 8.5	91.3 ± 12.3	<0.0001
Time to first mobilization (hours)	23.6 ± 6.3	32.9 ± 8.6	<0.0001
Time to first void after catheter removal (hours)	1.4 ± 0.8	1.3 ± 0.7	0.519
Length of hospital stay (days)	4.3 ± 1.1	6.7 ± 1.4	<0.0001

In the EFG, 31 participants (70.5%) reported being very satisfied compared to 19 participants (38.0%) in the TFG (p = 0.007). Additionally, nine participants (20.4%) in the EFG reported being satisfied, compared to 17 participants (34.0%) in the TFG. Neutral satisfaction levels were reported by four participants (9.1%) in the EFG and nine participants (18.0%) in the TFG. Notably, no participants in the EFG were dissatisfied, whereas five participants (10.0%) in the TFG reported being dissatisfied. No participants in either group reported being very dissatisfied (Table 3).

**Table 3:** Satisfaction levels between the early feeding group (EFG) and the traditional feeding group (TFG) VAS: Visual analog scale. Statistically significant for p < 0.05.

Satisfaction Level (VAS)	Early Feeding Group (EFG) (n = 44)	Traditional Feeding Group (TFG) (n = 50)	p-value
	Number (%)		
Very satisfied	31 (70.5%)	19 (38.0%)	0.007
Satisfied	9 (20.4%)	17 (34.0%)	
Neutral	4 (9.1%)	9 (18.0%)	
Dissatisfied	0 (0.0%)	5 (10.0%)	
Very dissatisfied	0 (0.0%)	0 (0.0%)	

The incidence of nausea was 9.1% in the EFG and 14.0% in the TFG (p = 0.461). Vomiting occurred in 6.8% of the EFG and 12.0% of the TFG (p = 0.394). Abdominal distension was reported by 9.1% of the EFG and 14.0% of the TFG (p = 0.461). Ileus occurred in 4.5% of the EFG and 8.0% of the TFG (p = 0.494). Wound infection rates were 4.5% in the EFG and 6.0% in the TFG (p = 0.753). Urinary tract infection was reported by 2.3% of the EFG and 4.0% of the TFG (p = 0.634). There was one case of deep vein thrombosis in the TFG (2.0%) and none in the EFG (p = 0.345). Readmissions occurred in 2.3% of the EFG and 6.0% of the TFG (p = 0.371). Overall, there were no statistically significant differences in the complication rates between the two groups (Table 4).

**Table 4:** Complication rates for the early feeding group (EFG) and the traditional feeding group (TFG) Statistically significant for p < 0.05.

Complications	Early Feeding Group (EFG) (n = 44)	Traditional Feeding Group (TFG) (n = 50)	p-value
	Number (%)		
Nausea	4 (9.1%)	7 (14.0%)	0.461
Vomiting	3 (6.8%)	6 (12.0%)	0.394
Abdominal distension	4 (9.1%)	7 (14.0%)	0.461
Ileus	2 (4.5%)	4 (8.0%)	0.494
Wound infection	2 (4.5%)	3 (6.0%)	0.753
Urinary tract infection	1 (2.3%)	2 (4.0%)	0.634
Deep vein thrombosis	0 (0.0%)	1 (2.0%)	0.345
Readmissions	1 (2.3%)	3 (6.0%)	0.371

## Discussion

The results of this study indicate that early feeding post-elective LSCS significantly improves recovery outcomes compared to traditional feeding protocols. The EFG demonstrated higher patient satisfaction, quicker return of bowel function, earlier mobilization, and shorter hospital stays.

Patient satisfaction

The VAS scores before discharge were significantly higher in the EFG (94.4 ± 8.7) compared to the TFG (81.4 ± 9.5), indicating greater patient satisfaction with early feeding. This result underscores the psychological and physical benefits of early nutrition, which can alleviate anxiety and discomfort associated with prolonged fasting [10]. These findings align with the study by Aydin et al., which reported improved patient comfort and satisfaction with early feeding following abdominal surgery [12]. Pravina et al. concluded that approximately 77% of the patients in the Enhanced Recovery After Surgery (ERAS) group rated the satisfaction score between 8 and 10 compared to 70% of the patients in the control group (p < 0.04) [13].

Time to first and second feed

The mean time to first feed in the EFG was 2.2 ± 0.8 hours compared to 23.9 ± 2.1 hours in the TFG, demonstrating a significantly quicker resumption of oral intake. Similarly, the time to second feed was also shorter in the EFG (10.4 ± 1.2 hours vs. 34.2 ± 3.4 hours). These findings support those of Pragatheeswarane et al., who found that early feeding led to faster recovery of gastrointestinal function [14]. The early introduction of oral intake stimulates the gut, promoting motility and facilitating a quicker return to normal bowel function [15].

Return of bowel function

The return of bowel function, measured by time to first flatus and stool, was significantly faster in the EFG. Patients in the EFG passed flatus at 23.9 ± 6.2 hours and stool at 54.6 ± 8.5 hours compared to 34.1 ± 8.8 hours and 91.3 ± 12.3 hours in the TFG, respectively. Early postoperative feeding is thought to stimulate gastrointestinal motility and promote a quicker return of bowel function. This is likely due to the early stimulation of the digestive tract and reduced postoperative ileus. These results are consistent with the physiological mechanisms discussed by Cusack et al., who noted that early feeding can mitigate the surgical stress response and support gastrointestinal recovery [16]. Pravina et al. reported a significant difference in the mobilization of patients of the two groups, but the difference in time to passage of the first flatus and feces was insignificant [13].

Time to first mobilization

The mean time to first mobilization was significantly shorter in the EFG (23.6 ± 6.3 hours) compared to the TFG (32.9 ± 8.6 hours). Early mobilization is crucial for reducing the risk of postoperative complications such as deep vein thrombosis and for promoting overall recovery. This finding aligns with the results of a study by Canzan et al., which reported that early feeding led to earlier ambulation and reduced hospital stays [17]. Enhanced mobility is associated with better pulmonary function, reduced risk of thromboembolism, and improved overall physical recovery [18,19].

Length of hospital stay

The length of hospital stay was significantly shorter for the EFG, averaging 4.3 ± 1.1 days compared to 6.7 ± 1.4 days in the TFG. Reduced hospital stay not only improves patient satisfaction but also has significant economic benefits by decreasing healthcare costs. These results are corroborated by the findings of Charoenkwan et al., who also reported shorter hospital stays with early feeding protocols [20]. Shorter hospital stays can reduce the risk of hospital-acquired infections and lower healthcare expenditures, benefiting both patients and healthcare systems. Baluku et al. reported that in cases of emergency cesarean section, a statistically significant shorter length of stay was found for the EFG as compared to the TFG, with a difference of 18.5 hours (p < 0.001) [21]. Mullman et al. stated that the length of stay from completion of the cesarean delivery to discharge decreased significantly post-implementation of the ERAS protocol [22].

Complication rates

Complication rates between the two groups were not significantly different, indicating that early feeding does not increase the risk of postoperative complications. The rates of nausea, vomiting, abdominal distension, ileus, wound infection, urinary tract infection, deep vein thrombosis, and readmissions were comparable between the EFG and TFG. This is consistent with the study by Teigen et al., which found no increase in complications with early feeding post-cesarean section [23]. The lack of significant differences in complication rates suggests that early feeding is a safe practice that does not compromise patient outcomes. McQueen et al. stated that the revolution of ERAS is sweeping high-income countries (HICs), improving surgical and anesthesia complication rates and outcomes, and saving healthcare expenses due to fewer complications and decreased length of stay in the hospital [24]. Similarly, a study by Charoenkwan et al. found that early feeding after cesarean section was safe and effective, with no increase in adverse outcomes [20].

Early oral intake stimulates gut hormones and the enteric nervous system, which in turn promotes peristalsis and reduces the duration of postoperative ileus. Moreover, early feeding helps maintain the integrity of the gut mucosa and prevents bacterial translocation, thereby reducing the risk of infections. The mechanisms described by Mundhra et al. highlighted how early nutritional intake can enhance recovery by supporting physiological functions essential for healing [25]. Several studies have reported similar benefits of early feeding in postoperative care. For instance, a study by Tamang et al. demonstrated that early feeding protocols were associated with faster recovery and shorter hospital stays across various surgical procedures [26].

Limitations

While this study provides valuable insights into the benefits of early feeding in elective LSCS patients, several limitations should be acknowledged. First, being a single-center study, the generalizability of the findings to other healthcare settings might be limited. Multicenter studies involving diverse patient populations are warranted to validate the results across different clinical contexts. Second, the sample size of this study may have limited the statistical power to detect small differences in outcomes between the EFG and TFG. Larger sample sizes would provide more robust evidence of the efficacy of early feeding protocols. Third, the follow-up period in this study was limited to 30 days postoperatively, and longer-term follow-up would be valuable to assess the sustainability of the observed benefits and monitor for late-onset complications or readmissions. Despite these limitations, this study contributes to the growing body of evidence supporting the implementation of early feeding protocols in elective LSCS patients, and future research addressing these limitations could further elucidate the optimal timing and duration of postoperative feeding regimens to optimize patient outcomes.

## Conclusions

While early feeding is only one of the components of ERAC, its application can improve patient satisfaction, recovery, and reduce the overall length of stay. This will ultimately reduce the unnecessary burden of cost of treatment and will help the healthcare of a developing country like ours. Future studies should continue to explore the long-term benefits and potential cost savings associated with early feeding strategies as well as the applicability of these findings to other surgical populations.
